# An integrative computational analysis provides evidence for *FBN1*-associated network deregulation in trisomy 21

**DOI:** 10.1242/bio.20134408

**Published:** 2013-06-20

**Authors:** Mireia Vilardell, Sergi Civit, Ralf Herwig

**Affiliations:** 1 Department of Vertebrate Genomics, Max-Planck-Institute for Molecular Genetics, Ihnestrasse 63–73, D-14195 Berlin, Germany; 2 Department of Statistics, University of Barcelona, Avenida Diagonal 645, Barcelona, 08028, Spain

**Keywords:** Down Syndrome, Marfan Syndrome, Cardiovascular, Heart, Bioinformatics

## Abstract

Although approximately 50% of Down Syndrome (DS) patients have heart abnormalities, they exhibit an overprotection against cardiac abnormalities related with the connective tissue, for example a lower risk of coronary artery disease. A recent study reported a case of a person affected by DS who carried mutations in *FBN1*, the gene causative for a connective tissue disorder called Marfan Syndrome (MFS). The fact that the person did not have any cardiac alterations suggested compensation effects due to DS. This observation is supported by a previous DS meta-analysis at the molecular level where we have found an overall upregulation of *FBN1* (which is usually downregulated in MFS). Additionally, that result was cross-validated with independent expression data from DS heart tissue. The aim of this work is to elucidate the role of *FBN1* in DS and to establish a molecular link to MFS and MFS-related syndromes using a computational approach. To reach that, we conducted different analytical approaches over two DS studies (our previous meta-analysis and independent expression data from DS heart tissue) and revealed expression alterations in the *FBN1* interaction network, in *FBN1* co-expressed genes and *FBN1*-related pathways. After merging the significant results from different datasets with a Bayesian approach, we prioritized 85 genes that were able to distinguish control from DS cases. We further found evidence for several of these genes (47%), such as *FBN1*, *DCN*, and *COL1A2*, being dysregulated in MFS and MFS-related diseases. Consequently, we further encourage the scientific community to take into account *FBN1* and its related network for the study of DS cardiovascular characteristics.

## Introduction

Down Syndrome (DS) is the most frequent autosomal aneuploidy that is compatible with post-natal life (1 per 700 newborns). It results from complete or partial trisomy of chromosome 21 (HSA21) and is characterized by a complex phenotype in which over 80 features occur with various degrees of expression and frequency causing a high inter-individual variability (Conti et al., 2007). Among them, and although DS is a major cause of congenital heart defects, there is a low risk of coronary artery disease (Vis et al., 2009a) which is classically related with athermanous plaques composed of macrophage cells, fatty deposits and fibrous connective tissue.

**Fig. 1.**
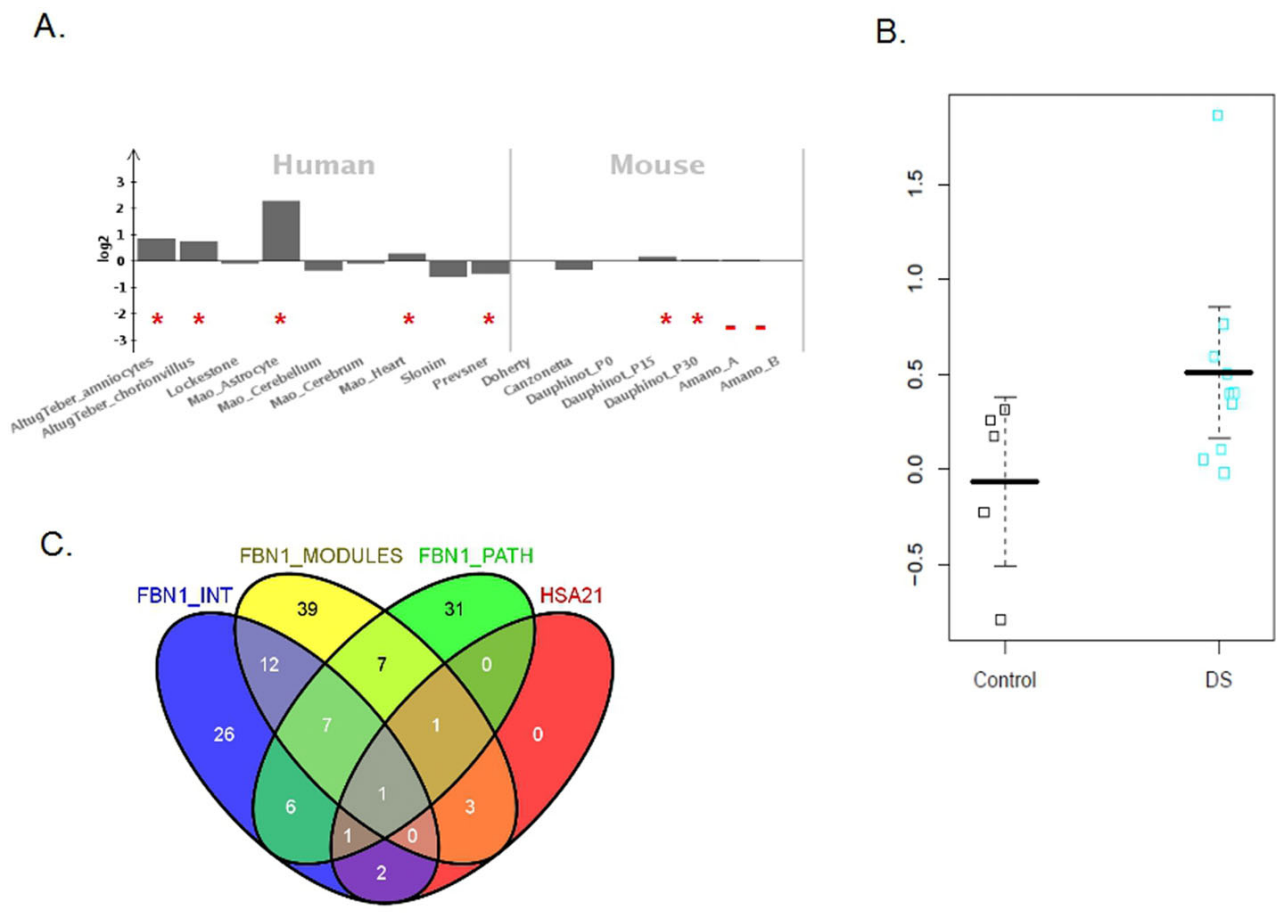
The role of *FBN1* and related genes in Down Syndrome. (**A**) The Meta-analysis results for *FBN1*. The major part of the studies with proved presence of expression are upregulated (red cruises *P*-value of presence <0.05, red dots *P*-value of presence <0.1). (**B**) Boxplot of deregulation of *FBN1* in DS heart. (**C**) Total number of significant in at least one study related to FBN1 neighbours (FBN1_INT), Functional Modules (FBN1_MODULES), related Pathways (FBN1_PATH) and genes on HSA21.

More than 200 genes on HSA21 could play a potential role in DS and, in spite of a lot of efforts of researchers worldwide, molecular causes of the main features remain still partially unknown. To gain more systematic insights in the molecular effects of DS, a meta-analysis on 45 different studies (Vilardell et al., 2011) was recently conducted and its results present a comprehensive resource for DS research with a catalogue of genes inside and outside of HSA21 being altered due to dosage effects, some of them highly related to other syndromes. One example is *FBN1*, a gene causative for MFS which was shown to be affected by dosage imbalance in DS (Fig. 1A). This finding is in concordance with earlier studies that suggested some overlapping features of DS with other syndromes (Ehler–Danlos) (Pasmatzi et al., 2006). Moreover, a recent paper proposed MFS compensating effects in DS, based on clinical manifestations, due to a case-report of a person affected by DS and MFS with moderate MFS visible (Vis et al., 2009b).

Marfan syndrome (MFS) is an autosomal dominantly inherited connective tissue disorder with an estimated prevalence of 1 or 2 per 10,000. It affects various organs, in particular the skeleton, the heart causing aortic dilation and the eyes, with variable phenotypic expression. MFS is induced by mutations in *FBN1* (Yao et al., 2007) and its haploinsufficiency as the main disease mechanism. This hypothesis is supported by the fact that a patient with a deletion that encompasses *FBN1* presents characteristic MFS (Hutchinson et al., 2003).

Although the molecular mechanisms that explain the interrelation of *FBN1* with MFS features are still unknown, there seems to be a pivotal role of the extracellular matrix components, encompassing some *FBN1* interactors (for example *MMPs* and *DCN*), which in some way could affect *TGFB* (upregulated) and/or its related pathways components linked to heart–vascular effects and BMP pathways linked to skeleton effects (Gao et al., 2010). Furthermore, effects of mutations in *TGFB2* or *TGFBR1*, *TGFBR2* lead to syndromes which share characteristics with MFS (Pezzini at al., 2012) (usually known as MFS-like syndromes) and highlight the importance of those pathways in MFS.

On the other hand, a recent study of DS in human heart samples reported as well alterations of extracellular matrix components, for example in *MMP2* and *COL6A2* (Conti et al., 2007).

The above findings are indicative of a plausible common dysregulation of certain genes in DS and MFS, maybe even with opposite trends, allowing compensation effects of the *FBN1* associated molecular network. *FBN1* network components are already found deregulated in other syndromes/diseases (Henrichsen et al., 2011; Mohamed et al., 2009) indicates their importance for understanding heart-related disorders and abnormalities.

The aim of this work is to conduct a comprehensive computational analysis at different levels (gene, network and pathway levels) in publicly available DS and heart tissue data in order to gain a better understanding of the *FBN1*-induced molecular network in DS heart tissue and its relationship with other genes and pathways. Additionally, we link the results with MFS and MFS-like disorders.

## Results

### Explanatory paragraph

To reach the objectives of this work, we have used a procedure devoted to identify genes potentially related to *FBN1* and tested their relevance in DS using publicly available DS microarrays datasets.

The results section is dived into five. In the first three sections we agglomerated a comprehensive list of genes related to *FBN1*; through analysis of its network interactors and related pathways and through the analysis of *FBN1*-coexpressed genes in heart tissue by using the Iterative Signature Analysis (ISA) method (Bergmann et al., 2003) (eisa: The Iterative Signature Algorithm for gene expression data, R package version 1.4.1, 2011, Gabor Csardi). Furthermore we have cross-validated those genes (see Materials and Methods) independently in two datasets: our own DS meta-analysis (Vilardell et al., 2011) performed with a large number of DS datasets (containing expression information of 19,389 genes) and a DS heart (tissue in which is proved *FBN1* expression, http://www.ebi.ac.uk/gxa/gene/ENSG00000166147) study (Conti et al., 2007) (containing expression information of 11,889 genes).

In the fourth section we have condensed the information from the three previous sections to a single numerical value with a Bayesian approach (see Materials and Methods). This gives a prioritization of genes with respect to their importance for DS context.

In the fifth section we have done a bibliographic revision of MFS or MFS-like studies including human and mouse models and we have analysed significant enrichment for MFS or MFS-like genes.

Finally, we have tried to identify a link between genes on HSA21 and *FBN1* in order to explain the effects of the molecular deregulation in DS patients.

### Aberrant expression of FBN1 and its interactors in DS

A previous meta-analysis which includes 45 independent studies provided a list of 324 genes whose expression was consistently altered in DS samples due to chromosome 21 dosage imbalances (Vilardell et al., 2011). Among the genes that had only little association with DS before we found *FBN1* which was predominantly upregulated in the studies under analysis (in 80% of the expression studies; upregulated in 4 out of 5 human studies and in 3 out of 5 DS mouse models (from the ones with a detection *P*-value <0.1; Fig. 1A). This upregulation was cross-validated with an additional dataset conducted with DS heart tissue (Conti et al., 2007) (Fig. 1B) after applying a Bayesian approach as described in Materials and Methods.

Abnormal expression of *FBN1* and its interactors have been reported before in heart diseases (Mohamed et al., 2009) and in connective tissue diseases (Henrichsen et al., 2011), providing an idea of their importance in those systems.

Since we were interested whether this trend of aberrant gene expression in DS would also hold for the interaction neighbourhood of *FBN1* we have investigated expression changes of *FBN1* and its interaction partners. A total of 249 direct and indirect network neighbours of *FBN1* were retrieved from databases (supplementary material Table S1). From those, 217 genes (from all 19,389 genes tested in the meta-analysis, see supplementary material Table S1) were interrogated finding 26 candidates in the meta-analysis (*P*-value = 9.9587e−13).

Furthermore, the DS heart study was used to cross-validate the meta-analysis results. From there, 207 genes that act as *FBN1* direct and indirect neighbours, were interrogated (from a total of N = 11,889 genes, see supplementary material Table S1) and 42 satisfies the inclusion criteria with an overlap of 13 genes from the meta-analysis (*P*-value = 4.396e−05). We have included for a posterior analysis 55 genes.

### FBN1-related pathways in the context of DS

We conducted Gene Set Enrichment Analysis (Subramanian et al., 2005) using the pathway information contained in ConsensusPathDB (Kamburov et al., 2011), version 12 (1,695 different pathways) and detected commonly altered pathways in the DS studies related to *FBN1* (identified as MFS pathway related can be seen in supplementary material Table S2; results in Table 1) which are fundamentally associated with the extracellular matrix. Taking into consideration only commonly deregulated pathways in both studies with FDR q-values <0.1, common alterations were found in: ECM-RECEPTOR INTERACTION, FOCAL ADHESION and INTEGRIN. We have also checked the INTEGRIN CELL SURFACE INTERACTIONS which is the unique pathway in ConsensusPathDB that contains *FBN1* as an integrand because of its clear significance in the meta-analysis and although the number of overlapping genes between both DS studies is high (Table 1) no significance was reached in the heart study.

**Table 1.**
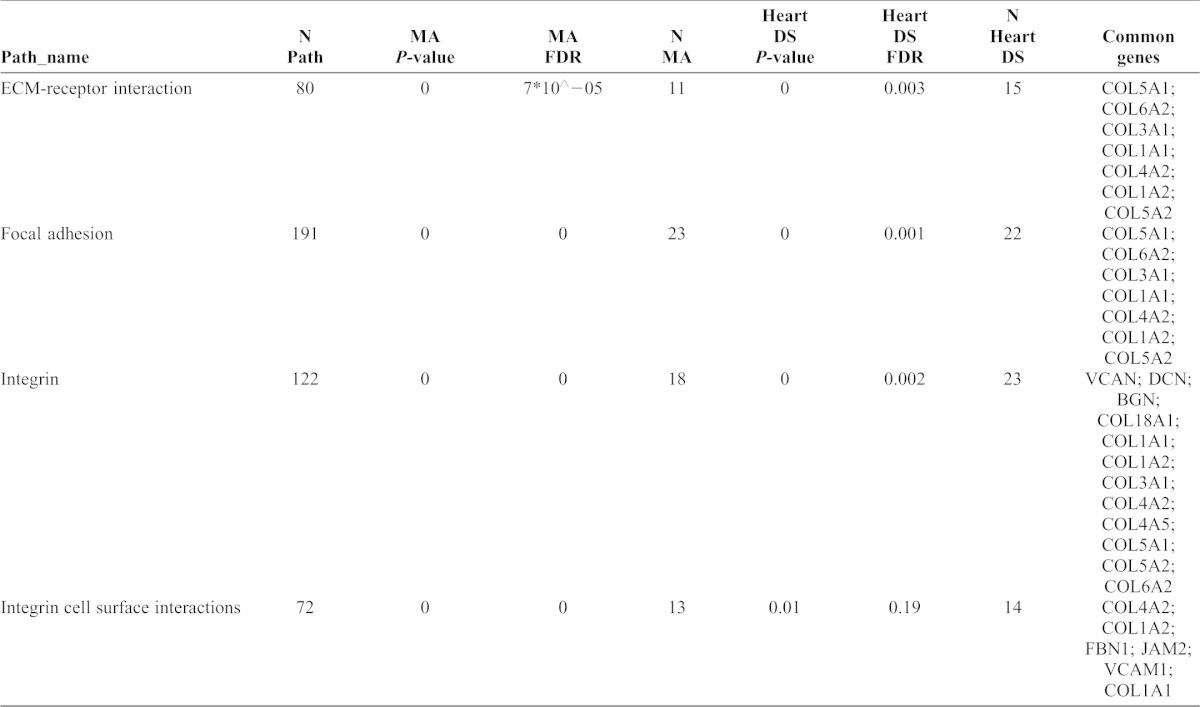
Pathways and related genes affected in the meta-analysis and/or in DS heart study. This table shows the number of genes in that pathway as compiled in ConsensusPathDB, the Gene Set Enrichment *P*-value from MA (Meta-analysis) and DS Heart study (Heart DS), FDR adjusted *P*-value, Number of Significant genes (N) for each study as well as the genes in common.

Thus, we can conclude that extracellular matrix components are deregulated in DS however no clear link with other related MFS pathways was found. By deeper examining the genes included in each pathway, we have found 54 genes from the pathway analysis that accomplish the inclusion criteria to further investigate their significance by using a Bayesian approach.

### FBN1 transcriptional co-expression module in human heart

In order to identify additional partners of *FBN1* to reinforce our knowledge of the system, we tried to identify co-expression modules centred at *FBN1*, i.e. genes that exert similar expression patterns across a set of experiments allowing getting extra information not conditioned by our actual knowledge of the system. To reach that, we have applied the Iterative Signature Algorithm (ISA) (Bergmann et al., 2003) (eisa: The Iterative Signature Algorithm for gene expression data, R package version 1.4.1, 2011, Gabor Csardi) (see Materials and Methods) that provides sets of co-expressed genes that are coherently either over- or underexpressed among samples, here also referred as functional modules.

We applied this algorithm to five independent Affymetrix heart studies not related with DS (supplementary material Table S3; see Materials and Methods). Using a stringent and unsupervised version of ISA, see Materials and Methods, we have selected two modules, which contained *FBN1* and are composed by 148 and 154 genes, respectively (supplementary material Table S4).

Using Gene Set Enrichment Analysis (Subramanian et al., 2005) with both modules, we have found a significant enrichment of both in the meta-analysis and in the heart DS study (FDR *P*-value <1*10^∧^−16). These two modules share more than 80% of the genes, because of that we have decided to merge them into a single one with final number of 181 genes and its overlapping between meta-analysis and DS heart study can be seen in Fig. 1C. Approximately 40% of these genes belong to the *FBN1* neighbourhood.

From the latest list of 181 genes, we found 43 candidates in the DS meta-analysis and 46 were found in the heart DS study with an overlap of 19 genes between both studies (Fig. 1C, *P*-value 0.0004). Consequently 70 were included to further study their significance.

### Integration of results with a Bayesian approach

A single commonality value that evaluates the concordance of the above results for each gene (from 136 unique genes extracted from the above sections, see Fig. 1C and supplementary material Table S5) was assigned using a Bayesian approach.

**Fig. 2.**
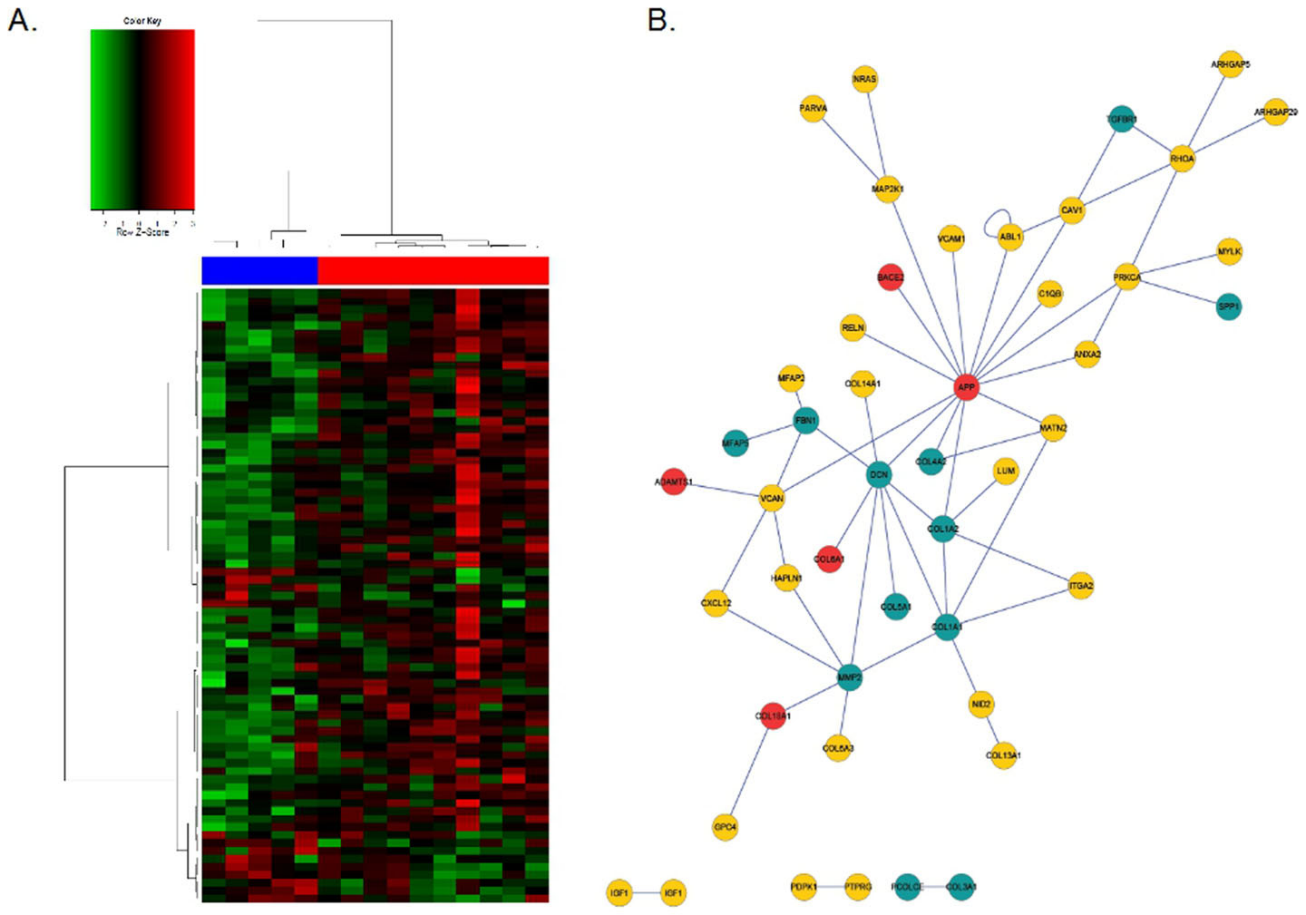
The role of *FBN1* and related genes in DS and in MFS. (**A**) Heatmap of the 77 genes with an absolute TBV greater than 2.58 from the Bayesian Analysis without genes on HSA21 (blue; control samples, red: DS samples). (**B**) Network reconstruction from all genes (N = 85) with an absolute TBV greater than 2.58 (red nodes; genes on HSA21, green nodes; MFS related genes).

This method resulted in 85 candidates, and after discarding genes located on HSA21 we have found a list of 77 regarding *FBN1* neighbourhood, functional modules or pathways which are able to discriminate control and DS samples (Fig. 2A) giving an additional proof that the *FBN1* system is altered in DS. Fig. 2B shows a network reconstruction using this set of genes.

Most of these genes have been previously related to heart morphogenesis, angiogenesis or atherosclerosis. Of special interest are the ones that can be associated to DS pathogenesis; among them we have found genes related to heart organogenesis (i.e. *VCAN*, which is essential for ventricular septal formation subsequent to cardiac atrioventricular cushion development (Hatano et al., 2012)), related to atherosclerosis or coronary artery diseases (i.e. *ANXA2*) (Seidah et al., 2012).

Two particular genes are also interesting; (i) Insulin-like growth factor (*IGF1*, upregulation in DS) which exerts multiple beneficial effects on the heart and can improve myocardial function in pathological situations (Touvron et al., 2012) and (ii) *NRAS* which is downregulated in the meta-analysis and also in the DS heart study. Mutations of this gene can produce juvenile myelomonocytic leukaemia (DS have a more risk than the normal population to suffer) or Noon syndrome that is characterized, mainly, by short stature and congenital heart disease (Kraoua et al., 2012).

### Common deregulation between DS and MFS

A completely independent systematic survey of MFS, MFS-like and their related mouse models from the PubMed database was done having as an inclusion criteria that the main topic of the study relates to the molecular mechanisms of those pathologies. We have included a total of 24 independent studies (supplementary material Table S6) and this provides a list of 325 candidate genes (supplementary material Table S7), however few of them have been suggested in more than one study (N = 52, 16%), indicating high degree of heterogeneity among studies and the necessity to make more efforts in order to decipher the main molecular mechanisms in MFS and related diseases. Consequently, to increase the consistency of the present study, we have decided to work only with the genes reported at least two times and call them MFS-related genes (N = 52, supplementary material Table S7).

First, we would like to challenge the gene search that we have done in relation to *FBN1*. We have interrogated the role of 1,485 candidates that belong to *FBN1* neighbours, functional modules or target pathways. From the list of genes related to MFS, 34 of 52 (65%) were evaluated in this study and 16 of them have become significant in the context of DS (47%, Fisher *P*-value = 1.63e−13).

From those genes, we would like to highlight the importance of *TGFRB1* (downregulated in DS samples), *LOX* and *DCN* (upregulated in DS samples) related to the *TGFB* pathway (Wang et al. described the potential role of those genes during heart development (Wang et al., 2005)). *ACTA2*, collagens as well as *FBN1* and *MMP2* are associated to extracellular matrix producing different types of heart abnormalities.

Additionally, the network reconstruction highlight the importance of HSA21 genes in relation this deregulation (Fig. 2B) suggesting different pattern of deregulation in DS patients than in MFS. Therefore, MFS related genes (represented in green) are not allocated randomly, rather are located specifically around *FBN1* and *DCN*. Deregulation of the tandem *DCN-FBN1* is classically observed in MFS and other syndromes like Williams–Beuren (Henrichsen et al., 2011).

### Role of HSA21 genes

The final gene priorization list contains 8 genes located on HSA21 (supplementary material Table S5); *ADAMTS1*, *ADAMTS5*, *APP*, *BACE2*, *PIGP*, *COL6A1*, *COL6A2* and *COL18A1*. In order to know which of them could explain the observable *FBN1* deregulation in DS, we have tried to correlate the expression of those HSA21 genes with *FBN1* in the meta-analysis and in DS heart study (note that co-expression with *FBN1* comes from independent studies) finding that the top three genes with greater positive Pearson correlation and higher concordance between studies were found for *BACE2*, *COL18A1* and *COL6A2* (Table 2). From them *COL6A2* and *COL18A1* are of special interest because they belong to the classical critical heart region for DS (Barlow et al., 2001).

**Table 2.**
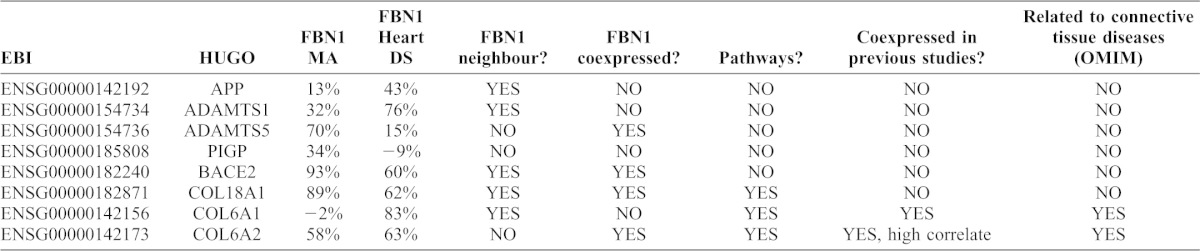
*FBN1* correlations with HSA21 candidates.

In spite of having evidence that *BACE2* is expressed in heart, its relation to *FBN1* is not clear. Nevertheless, *COL6A2*, which is part of a protein complex, additionally, by *COL6A1* and *COL6A3* (*FBN1* neighbours, see Fig. 2B), has been proposed in one study as a candidate to modify the phenotype expression of MFS (Summers et al., 2010).

## Discussion

In this work, we provided an integrative DS study and analysed the role of the *FBN1*-network in DS heart tissue. This study was motivated by two other recent studies that suggested *FBN1* alterations in DS (Vilardell et al., 2011; Pasmatzi et al., 2006; Vis et al., 2009b) which is usually downregulated in MFS, MFS-like (Gao et al., 2010) and in other connective tissues disorders like Williams–Beuren syndrome (Henrichsen et al., 2011). Its importance seems to be crucial for the development of heart and the aortic system (Gao et al., 2010).

The current study relies on the integration of different sources of data and on a two-step statistical analysis which combines standard statistics and a Bayesian approach (Results section). The Bayesian approach takes advantage of the results of a previous meta-analysis study in DS (Vilardell et al., 2011) allowing to build a normal prior distribution for each gene based on previous experimentation (see Materials and Methods). Prior choice based on previous analysis, particularly meta-analysis, has been emerging as a reliable and powerful tool in Bayesian statistics (Congdon, 2006).

Thus, in a first step we have analysed two studies related to DS and after applying, independently, standard statistical tests we have found evidence for *FBN1* network regulation in both studies encompassing 136 candidates genes. Furthermore, using the pathway information of ConsensusPathDB and performing Gene Set Enrichment analysis, we have found evidences of an enrichment of affected genes related to the Extracellular Matrix in DS studies (ECM-RECEPTOR INTERACTION, FOCAL ADHESION, INTEGRIN and INTEGRIN CELL SURFACE INTERACTIONS). Extracellular Matrix Components have been associated before to heart malformations (Hinton and Yutzey, 2011) and to coronary artery risk (Raffetto and Khalil, 2008; Bench et al., 2011) in non DS studies which is consistent with DS features.

On the other hand, after applying an integrative Bayesian strategy over the 136 candidates, we have obtained 77 genes outside HSA21 able to classify control and DS samples (Fig. 2A). Those genes are mostly upregulated, however down regulation trends can also be observable (i.e. *TGFBR1*).

Additionally we did a survey of MFS and MFS-like diseases and we have found that our DS data is consistent with the presence of a certain overlap between MFS or MFS-like gene deregulation (47%) leading the extracellular matrix components. Remarkable is this last result highlighting genes like *MMP2*, *COL1A1* and *COL1A2* previously validated by PCR in the DS heart study from Conti et al. (Conti et al., 2007) and *FBN1*, *DCN*, *COL1A2*, *COL1A1* and *COL3A1* significant in the meta-analysis and cross-validated in the DS heart study (Conti et al., 2007) through the Bayesian approach.

Finally, in order to try to explain how genes on HSA21 can modify expression of *FBN1* and other related genes, we looked at the direct *FBN1* molecular interactors and we found 8 genes on HSA21 that remain significant after the application of the Bayesian approach finding three of them with stronger evidence for a related role; *BACE2*, *COL18A1* and *COL6A2*. Of these *COL6A2* showed the highest evidence for being causative of that deregulation; the gene has an additional role to *FBN1*-related pathways, it is mentioned as highly correlating gene together with *FBN1* in a previous study, has been related before with DS heart features (integrand of the critical DS heart region), participates in a complex that links indirectly with *FBN1* (*COL6A3* through *MMP2*, *COL6A1* through *DCN*) and its deficiency is related with a connective tissue disease, however, without any evident role in heart.

Thus, this survey proposes a new list of gene candidates related to DS, some of them display similar molecular mechanism affected in DS and in MFS mostly related to the extracellular matrix, with one or more genes on HSA21 responsible for that fact (from which *COL6A2* appeared the most plausible candidate). The repercussion of the alteration of that system in DS disease can be related with the special cardiovascular characteristics of DS patients being either the higher risk of developing heart abnormalities, in special related to valve formation (Hinton and Yutzey, 2011), or the overprotection against coronary artery disease (found in some epidemiological age-matched studies (Ylä-Herttuala et al., 1989; Murdoch et al., 1977)). Deregulation of genes like *VCAN* (Hatano et al., 2012), *LOX*, *ACTA2* and *MMP2*, related with heart development (Hinton and Yutzey, 2011), are good candidates to explain the higher risk of heart abnormalities in DS. Otherwise, key genes have been emerged through our analysis related to the formation of the atherosclerosis plaque affected in DS, i.e. upregulation of *ANXA2* (Seidah et al., 2012), although other factors like sex hormones (El Khoudary et al., 2012; Mendelsohn and Karas, 2005; Suzuki et al., 2010) can also be related to this feature.

Taking all those findings together, we consider that the deregulation of *FBN1*-associated network could be crucial to understand the cardiovascular characteristics associated with DS as it is in other syndromes (Henrichsen et al., 2011; Mohamed et al., 2009). However further validation and functional studies are still necessary to assess its importance in the DS context.

## Materials and Methods

### Expression data, standardization and normalization

Reviews and quantitative expression data for either DS or MFS were collected from PubMed, ArrayExpress (Kapushesky et al., 2012) and Gene Omnibus Express (Barrett et al., 2011) databases.

Two DS datasets were used; the results from a previous meta-analysis (Vilardell et al., 2011) where a total of 19,389 genes across 45 different studies were tested and additional independent gene expression data (Affymetrix U133A platform; GSE1789) on heart tissue from human DS cases composed by 10 DS foetuses samples (5 DS without cardiac abnormalities and 5 DS displaying different heart defects) and 5 controls (non DS foetuses without heart abnormalities) (Conti et al., 2007). The latter dataset allowed cross-validating the meta-analysis results which were mainly based on DS brain in the context of DS heart tissue. Affymetrix oligoprobes were remapped to the human genome using Ensembl (version 56) annotation which results in 11,889 informative genes. An overall control mean for each gene was computed and log2 ratios for each sample with respect to that control value were calculated (standardization).

Furthermore, five additional gene expression datasets generated from normal human heart tissue (Affymetrix U133A platform) were used for analysis of functional modules. All data were normalized using the GCRMA method. Probes were re-annotated as described above.

### Statistical analysis

To determine statistical significance in DS studies a Bayesian strategy was used over a set of candidates previously identified using two approaches, based on standard statistical analysis, due to their different nature. First, candidates from the meta-analysis were included using a non-stringent score cut-off of 3.4 (scores were obtained as described (Vilardell et al., 2011)). Second, to determine candidates from the DS heart study (Conti et al., 2007) a shrinkage student's t-test (Zuber and Strimmer, 2009) was used (DS samples versus controls) and genes with *P*-values <0.1 were considered for further testing their significance using a Bayesian Approach. To determine whether a certain overlap between studies was present, a Fisher's test was conducted and significance was considered for *P*-values <0.05.

Bayesian methods are based on Bayes theorem (see below; Bayes theorem in Probability) providing tremendous flexibility for data analytic models and yield rich information about parameters that can be used cumulatively across progressive experiments being special useful when it is known some qualities from the data before (allowing to build a prior distribution). In this situation provides robustness in front of small sample sizes by using parametric distributions.

Moreover, this methodology provides us a unique posterior distribution from different kinds of studies which can be summarized by the estimates of its parameters and obtained a unique value for each gene (here referred as Typified Bayes Value, TBV, see below; Interpretation of the Bayesian Values).

The method adds a major weight of the observations accounted in DS heart study if they are not clearly contradicted by the meta-analysis results taking into consideration the expected and natural expression changes that can be measured. The method needs, in a first step, a prior knowledge about the possible range of values for each gene and this was performed using the log2 fold-change mean from the studies considered in the meta-analysis and its standard error from 16 arrays experiments with expressed *P*-values <0.1.

Since the most extended way to analyse log2 ratios from microarray studies are based on normal distribution (Allison et al., 2006; Dobbin and Simon, 2005) and the fact that independent means follows a Normal distribution by the application of the Central Limit Theorem we have consider a Normal Distribution for the group of means obtained from each microarray study.

In a further step, we evaluated the DS samples from the DS heart study and, following the same reasoning, we consider a normal distribution for the log2 ratios in the Conti study (each Down Syndrome samples versus the mean of the Controls samples) which is additionally the most conservative alternative (Albert, 2007) (see below; Prior distribution, sampling distribution (likelihood), Bayes' rule, Posterior distribution) and keep the coherence with the previous Conti analysis. Finally, we summarized the posterior distribution by calculating a statistic based on its posterior mean divided by its posterior standard error (Typified Bayes Value, TBV, see below; Interpretation of the Bayesian Values). The genes that remind significant (absolute TBV>2.58) were visualized with heatmaps which group together samples (columns) and genes (rows) which display a similar pattern through hierarchical clustering. Dendrograms were built using the Canberra distance as a distance metric and a complete linkage as agglomerative method. Finally, in order to correlate expression profiles of genes among either the meta-analysis or the DS heart study the Pearson correlation was used.

#### The Bayes theorem in probability

The Bayes theorem can be written as follows:
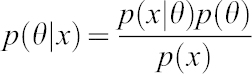

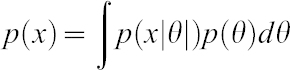
By formulating a prior belief of the probability distribution of the parameter of interest, θ, (*p*(*θ*)) and by replacing 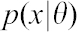 with the corresponding likelihood function of the observations, we can rewrite as follows:
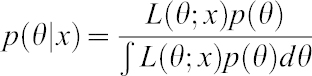
which is the posterior distribution probability for θ once we have observed the data and conjugate with our prior belief as it is represented above.

#### Prior distribution, sampling distribution (likelihood), Bayes' rule, posterior distribution

A prior is often the purely subjective assessment of an experienced expert or results from previous studies (Casella and Berger, 1990). We have used a previous meta-analysis composed by 16 independent microarrays studies to build the prior distribution.

From each study a log2 ratio mean was computed if the *P*-value of gene expression was below 0.1 (due to the way that the selection was performed in the Meta-Analysis only widespread expressed genes were tested).

Then, if we consider a Normal prior for the parameter θ, Ν(θ_0_,σ^2^_0_) as described (Albert, 2007; Casella and Berger, 1990):
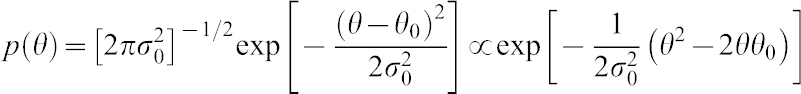
(where θ_0_ is estimated as the mean of the log2 ratios means from each study and σ_0_ is estimated as the standard deviation of these means) and a normal likelihood for the observed data (N(θ,σ^2^)):
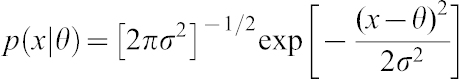

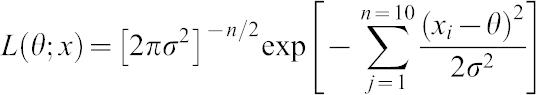
then the posterior follows also a Normal distribution with parameters N(θ_1_,σ^2^_1_) and can be written as follows:
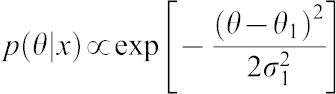
The posterior parameters can be estimated as follows:
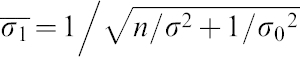

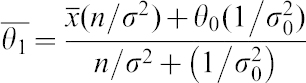
where θ_0_ and σ_0_ come from the prior Normal Distribution and σ has been estimated from the standard deviation of the data (Conti et al., 2007) and *n* is the sample size (in this case 10 Down Syndrome cases).

#### Interpretation of the Bayesian values

After getting the estimated of θ_1_,σ_1_ for each gene *i* we can consider the following statistics which we call Typified Bayes Value (TBV):
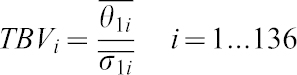
If TBV follows a normal distribution, then absolute TBV greater than 2.58 falls outside of the 99% probability of being θ_1_ = 0.

### Bioinformatics analysis

*FBN1* network neighbours were determined using the protein–protein interaction information from ConsensusPathDB version12 (Kamburov et al., 2011) which contains a large number of molecular interactions of different types.

Functional Modules were identified using the Iterative Signature Algorithm (ISA) embedded in the eisa library of the Bioconductor software, as described (Bergmann et al., 2003) (eisa: The Iterative Signature Algorithm for gene expression data, R package version 1.4.1, 2011, Gabor Csardi), after filtering for genes with a detection *P*-value <0.1 in at least l% of the samples and with thresholds 2, 2.2, 2.4, 2.5 for samples and features. From these modules we selected 2 modules from which the co-expression of the genes was observed in more than 5 samples (to increase the robustness of the analysis).

Network reconstruction was realized in Cytoscape using the interactome provided by ConsensusPathDB version 12 and Venn diagrams were created by VENNY software (VENNY, an interactive tool for comparing lists with Venn diagrams, 2007, Juan C. Oliveros).

Gene Set Enrichment Analysis (Subramanian et al., 2005) was performed independently over the whole list of genes from DS meta-analysis (19,389) and the whole list of genes from the DS heart study (11,889) in order to discern whether an enrichment of functional modules (ISA) or pathways from the ConsensusPathDB was present. Significance was considered for a pathway or functional module with FDR q-value <0.05.

All bioinformatic analyses were conducted in R and associated libraries available from Bioconductor or cran repositories.

## Supplementary Material

Supplementary Material
