# Supplementary material for: An integrative computational analysis provides evidence for FBN1-associated network deregulation in trisomy 21
Source: Biol Open. 2013 Jun 20;2(8):771–8. doi: 10.1242/bio.20134408 (PMC3744068; doi:10.1242/bio.20134408)
Supplement: Supplementary Material [file supp_2_8_771__index.html]

An integrative computational analysis provides evidence for FBN1-associated network deregulation in trisomy 21 — An integrative computational analysis provides evidence for FBN1-associated network deregulation in trisomy 21 — Supplementary Material 

# An integrative computational analysis provides evidence for *FBN1*-associated network deregulation in trisomy 21

## 

**Files in this Data Supplement:**

- Supplementary Tables S1-S7
